# NINQ: Name-Integrated Query Framework for Named-Data Networking of Things

**DOI:** 10.3390/s19132906

**Published:** 2019-06-30

**Authors:** Muhammad Atif Ur Rehman, Rehmat Ullah, Byung Seo Kim

**Affiliations:** 1 Department of Electronics & Computer Engineering, Hongik University, Sejong City 30016, Korea; 2 Department of Software and Communications Engineering, Hongik University, Sejong City 30016, Korea

**Keywords:** Named-Data Networking, internet of things, query, commands, smart building, Pull and Push Support

## Abstract

Information-Centric Networking (ICN) is a paradigm shift from host-to-host Internet Protocol (IP)-based communication to content-based communication. In ICN, the content-retrieval process employs names that are given through different naming schemes such as hierarchical, flat, attribute, and hybrid. Among different ICN architectures, Named-Data Networking (NDN) has gained much interest in the research community and is actively being explored for the Internet of Things (IoT) and sensor networks, and follows a hierarchical naming format. NDN protocol follows a pull-based communication model where the content consumer gets content irrespective of the location of the content provider. The content provider in NDN and sensor networks can be considered to be a distributed database that monitors or controls the environment and caches the sensed data or controls information into their memory. The proposed Name-INtegrated Query (NINQ) framework for NDN-based IoT provides a flexible, expressive, and secure query mechanism that supports content retrieval as well as control and configuration command exchange among various nodes in a smart building. Different use cases are presented in this paper that expand on the behavior of proposed query framework in different scenarios. Simulation results of data collection and exchange of control commands show that proposed query framework significantly improves Interest Satisfaction Rate (ISR), Command Satisfaction Rate (CSR), energy efficiency, and average delay. Moreover, it is evident from the simulation results that proposed query framework significantly reduces the number of transmissions in the network in both data collection and exchange of control command scenarios, which improves the network performance.

## 1. Introduction

In traditional Internet architecture, communication between a client and a server occurs once a stable connection comprising two client-side steps has been established. These steps are (1) the translation of a user-friendly hierarchical URL to the IP address of a host machine using Domain Name System (DNS) lookup, and (2) forwarding the request to the server machine whose IP address is obtained in step one. This Internet architecture was designed for client–server applications such as Hypertext Transfer Protocol (HTTP), File Transfer Protocol (FTP), and Simple Mail Transfer Protocol (SMTP) to cope with the early Internet need for location-aware scarce resource sharing. However, today’s Internet has evolved from this host-oriented communication to a more content-based model instead. As reported in the Cisco Visual Networking Index 2017–2022 [1], 82% of Internet traffic in 2022 will be video content and 72% will be carried by Content Delivery Networks (CDNs). To satisfy these huge content-related requirements, incremental patches such as DNS, CDNs, and peer-to-peer (p-to-p) content sharing have been introduced. The core functionality of DNS resolves content names to content location (IP addresses). However, CDN and p-to-p significantly improves the content-retrieval process by bringing the content closer to the end user. However, still they are an overlay on existing traditional IP-based location-aware Internet architecture.

The paradigm shift in user demand and the limitations of existing architecture have motivated researchers to look for alternative solutions for the future Internet with Information-Centric Networking (ICN) [2] appearing as promising candidate. There have been many architectures proposed under the ICN umbrella, such as Named-Data Networking (NDN) [3] which has gained much interest in the research community and is actively being explored. NDN treats names as a first-class entity and adopts a pull-based consumer-driven communication model where the consumer’s request for content is forwarded by a content router (CR) if not already cached in the content store (CS) of the CR. The Interest packet then reaches a content provider which responds with a Data packet sent back to the consumer along a breadcrumb path that is created with the help of a Pending Interest Table (PIT). NDN has an adaptive forwarding mechanism and employs a Forwarding Information Base (FIB) table that retains information for forwarding planes.

Another fundamental difference between IP and ICN/NDN is the stateless and stateful nature of their CRs, or relay nodes. In IP, since the nodes are stateless, they are not aware of the content of the packets routing through them and do not cache the content in their memory. However, in NDN, the nodes are stateful; they are aware of the content that passes through them and cache Data packet content so that the same content can be provided quickly for future demands. Thus, NDN uses its routers as storage devices, and the interconnection of these routers can be considered a distributed database.

The content of such a distributed database can be homogeneous and/or heterogeneous in nature, particularly when a network comprises sensor nodes or devices associated with the Internet of Things (IoT). NDN uses a hierarchical naming scheme to fetch the desired content from a network. However, this naming scheme is not fully compatible with IoT applications or a network that comprises sensor nodes because the contents of the networks can be highly dynamic and changeable. Moreover, IoT devices may also exchange control commands with each other which is not fundamentally supported in conventional NDN naming schemes. Dealing with this situation by using legacy naming schemes creates a lot of unnecessary packet transmission in the network and, as a result, decreases Interest and command satisfaction rates (ISR and CSR, respectively), increases overall round-trip time, and increases the chances of congestion in the network.

To address these limitations, this paper proposes a name-based query framework, the Name-INtegrated Query (NINQ), which is an overlay on existing NDN architecture and provides an efficient query mechanism to extract heterogeneous content from the nodes. Even though name resolution in the proposed framework follows a conventional mechanism, once the name is resolved, a consumer-driven query is evaluated to filter the content. Moreover, the NINQ framework also provides a secure and reliable mechanism to exchange control commands between different nodes on the network.

The contributions of the proposed NINQ framework are as follows:

1. The NINQ framework provides a hybrid naming scheme for NDN that incorporate the hierarchical name components, hash-based flat part, and flexible, expressive, and secure query part to gather the heterogeneous data from the IoT devices.

2. The NINQ framework introduces a sub-part in the content name to support the execution of configuration or control commands on IoT devices with the help of different constraints.

3. NINQ framework enables efficient push-based unsolicited data packet transfer to the appropriate consumer(s) to support periodic and event-driven communication in a network.

4. The NINQ framework employs a flat component to secure its query, constraint, and command components;

5. We consider a smart building equipped with heterogeneous IoT devices such as a thermostat, smoke detectors, and smart air cleaner, to evaluate the proposed query framework. The simulation results of query-based data collection and exchange of action commands between these heterogeneous devices show that the proposed framework significantly outperforms the most recent naming schemes in terms of ISR, CSR, average delay, and energy consumption, and also reduces the number of Interest and Data packet transmissions.

We believe that the applicability of the proposed NINQ framework is not limited to smart buildings, and that it could be used for other IoT applications such as personal health and wellness, smart vehicular networks, and wireless sensor networks.

The rest of this paper is organized as follows: Section 2 provides a brief overview of the IoT and NDN, and Section 3 presents the most recent related work. A motivation with the help of communication service models in IoT-based smart building is presented in Section 4, while a detailed description of the proposed NINQ framework is described in Section 5. The simulation scenario, results, and discussion are presented in Section 6. Subsequently, we present some of the open research challenges and future directions of the NINQ framework in Section 7 and, finally, we conclude the study in Section 8.

## 2. Internet of Things and Named-Data Networking (in a Nutshell)

The IoT is a vision of connecting all ‘things’ from every aspect of life including smart phones, household electronic equipment, smart vehicles [4], the smart electrical grid [5], smart home [6,7], smart campus [8,9], monitoring and gathering data from sensors or actuators deployed in agricultural contexts [10], and RFID tags [11] through a different set of networking technologies. The IoT is content-centric, and, most of the time, its users are more interested in getting updated content rather than the location of that content [12]. IoT devices often produce content on a massive scale and/or exchange control commands to perform management tasks. For example, IoT devices in the field of wireless sensor networks (WSNs) do specific tasks such as gleaning raw data or useful information on a large scale from their surroundings; each sensor node performs its assigned task, for instance a humidity sensor only measures moisture in the air whereas a temperature sensor will only measure the temperature of its environment. Furthermore, IoT devices in a building management system often exchange control information, for example, by using a centralized controller (CC) that can forward turn-on commands to the air conditioner (AC) in a specific room based on the temperature information it has received.

There are many practical challenges in the IoT that need to be addressed such as handling the dynamic changes in the network, enabling communication between heterogenous and constrained devices, and maintaining consistent and secure communication across the network [13]. The IoT devices are still using conventional host-centric IP-based Internet architecture which is not suitable for them [13] because they are often resource-constrained in terms of computation, communication, and storage, or are deployed in inauspicious environments, sometimes buried under the ground or underwater, with intermittent connectivity that makes it harder to sustain a stable connection. To solve such issues in the current IoT era, NDN has appeared as a promising solution.

NDN follows a hierarchal human-readable naming format with a sequence of components separated by “/”. This hierarchal system provides aggregation of content names in a routing table which potentially leads to a smaller routing table and reduces router storage and computation requirements. In NDN, content name is assigned by the producer of the content and this helps in forwarding the Interest and Data packets between the consumer and producer and in caching the content at intermediate relay nodes. The NDN naming format is similar to URLs, for example “http://www.hongik.ac.kr/” can be represented as “ndn:hongik/ac/kr”. In terms of the IoT, the CC in a smart building (detailed discussion of which is presented in Section 4) may issue an Interest packet carrying a hierarchical name such as “ndn:hongik/sejong/d/4/425/temperature” to request the current temperature from the thermometer installed in room 425 on the fourth floor of D building at Hongik University Sejong Campus. The thermometer may then respond to this request with a Data packet that contains the current temperature value in that room. After receiving the content, the CC forwards another Interest packet (command and/or constraints) to take the appropriate action to adjust the AC settings.

**Figure 1:**
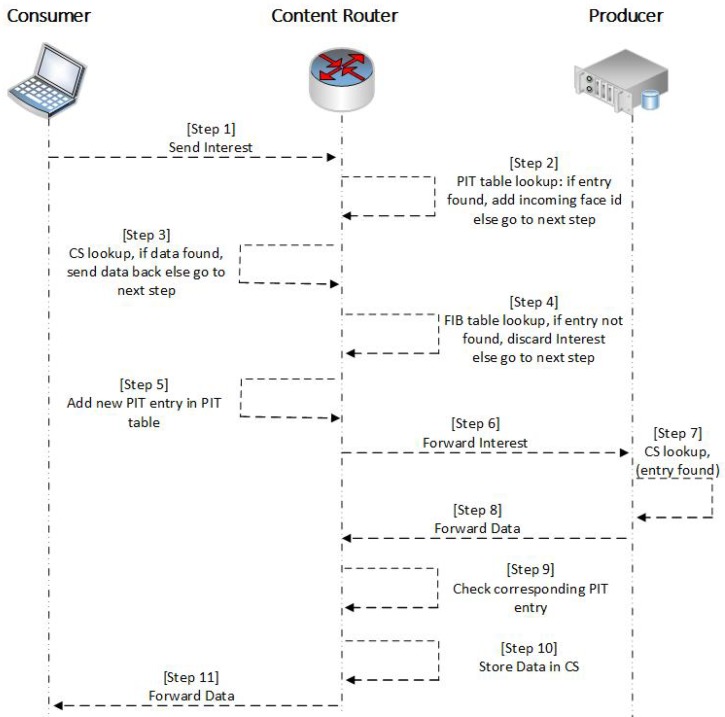
NDN communication process.

In NDN, each node maintains three types of data structure: (1) a PIT; (2) a CS; and (3) FIB. Figure 1 presents the NDN communication process in a network. When a consumer node wants to access specific content, it inserts the name of the desired content into an Interest packet and forwards it to the network. When the CR receives the Interest packet, it first checks the PIT entries to check whether a request for the same content is already in the queue. If the CR has a matching PIT entry, it simply stores the incoming face ID of the current request. In the absence of a PIT entry, it checks its own CS for the desired content; if a match is found, the CR responds with the Data packet through the interface from which the Interest arrived. However, if the desired content is not present in the CS, the CR forwards the Interest packet to the producer using the longest prefix matched on the FIB and inserts a new entry in the PIT table. The producer node only responds with a Data packet once it receives the Interest packet. The Data packet comprises the name of the content, the content itself, and other optional fields. When the Data packet arrives at the CR, it searches the corresponding PIT entry and forwards the data to all downstream face IDs. Moreover, the CR stores the data in its CS to satisfy any future requests for the same data without having to fetch it from the producer. Finally, the CR removes the corresponding entry from the PIT table.

## 3. Related Work

IoT devices produce content on a massive scale, and it is expected that billions of data may be produced in a single second. This makes heterogeneity and scalability a fundamental requirement of IoT architecture, but to meet these requirements with existing TCP/IP solutions is extremely complex and inefficient [13].

**Figure 2:**
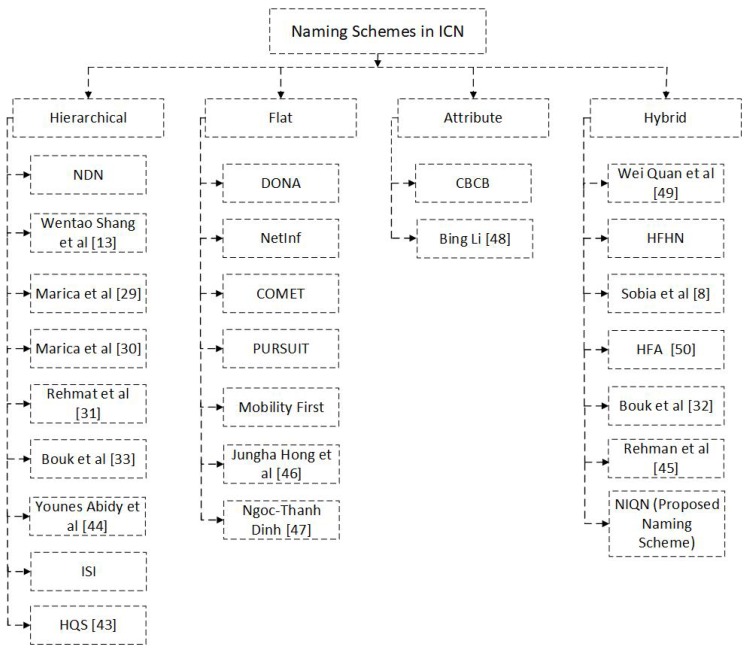
Naming Schemes in ICN.

The ICN on the other hand, with the help of its naming techniques may meet the aforementioned IoT requirements. There are four types of naming techniques outlined in the ICN literature (Figure 2): hierarchical, flat, hybrid, and attribute. In hierarchical naming, names are given in plain and human-readable components, separated by “/”. The most prominent ICN architectures, CCN and NDN, follow this hierarchical naming convention. Flat names are self-certifying and used in ICN architectures such as PSIRP [14], PURSUIT [15], MobilityFirst [16], DONA [17], NetInf [18], COMET [19], CONVERGENCE [20] and SAIL [21]. The attribute-based technique was first introduced in Combined Broadcast and Content-Based [22] routing, and hybrid naming combines all these other three techniques [23,24]. In the ICN literature about the IoT, most research focuses on and explores hierarchical [13,25,26,27,28,29,30,31,32,33,34,35,36] and hybrid [8,9,37,38,39,40] naming specifications. Flat [41,42] and attribute-based [43] naming techniques are beyond the scope of this paper and so the related work only covers the hierarchical and hybrid approaches which are most relevant to the smart building scenario and follow NDN protocols.

Sobia et al. [8] introduce a hierarchical and flat-based hybrid naming (HFHN) scheme for smart buildings.

The hierarchical components of the Interest packet consist of the domain name, location, and task, while the flat component is the hash of the device name. The payload in the Data packet is secured by taking the cryptographic hash of its value. In this study, the authors evaluated their naming scheme by considering both static and mobile nodes in the network. However, they only increased the number of nodes and did not increase the node speed which greatly affects the collection of transient data from IoT devices. Moreover, the authors did not provide a query structure for such transient data collection.

In [31], a push-based data broadcast control naming scheme for smart buildings is proposed. The scheme consists of a hierarchical namespace for contents, a hierarchical namespace for IoT devices, and minor modifications to Data packets to support emergency scenarios. This naming scheme only supports a push mechanism and lacks pull-based communication which is a fundamental requirement for the IoT. Moreover, this study only considers static nodes in the network. Elsewhere, [30] propose a pull-based hierarchical naming framework for an IoT smart home with a scheme that consists of root/homeID, task class, task type, task sub-type, and location. This proposed naming scheme covers both action and sensing tasks; however, it does not provide any security mechanism for the critical sub-components of task type, task sub-type, and location. Moreover, the proposed pull mechanism for data collection is inefficient for transient IoT content and creates unnecessary packet transmission in the network.

The scheme proposed in [37] only considers mobile nodes and was specifically designed for vehicular ad hoc networks. Moreover, the naming scheme here does not provide a query mechanism for efficient data extraction from the network. Bouk et al. [32] propose a further hierarchical naming scheme, this time for underwater sensor networks, that consists of time, location, sensed data type, and preference components. The authors of this study only consider pull-based communication between the underwater sensor nodes and, in addition, their naming scheme does not provide any mechanism to secure the critical components of time, location, and preference. Similarly, Marica et al. [33] do not secure the service name which constitutes the most important component in their naming scheme for both content and services to process large amounts of IoT data at the network edge. Instead, the tag IoTNCN is used to distinguish content from service names. Moreover, this study also only considered static nodes in the network which limits its applicability in mobile scenarios.

In summary, these naming schemes try to address issues of communication between IoT devices. However, most lack either one or all core IoT requirements: (1) they do not consider mobility and security in the proposed schemes; and/or (2) they do not provide an efficient query structure to extract transient IoT data; (3) they do not support action/configuration command exchange among IoT devices. In contrast, our proposed name-based query mechanism not only includes hierarchical components but also a flat element to secure the Interest and Data packets, querying logic as the query component to extract data from the transient IoT, and a command and/or constraint component to take appropriate action on a node. A summary comparison of our proposed naming scheme with other recent proposals is presented in Table 1.

## 4. Motivation: A Smart Building Use Case

**Figure 3:**
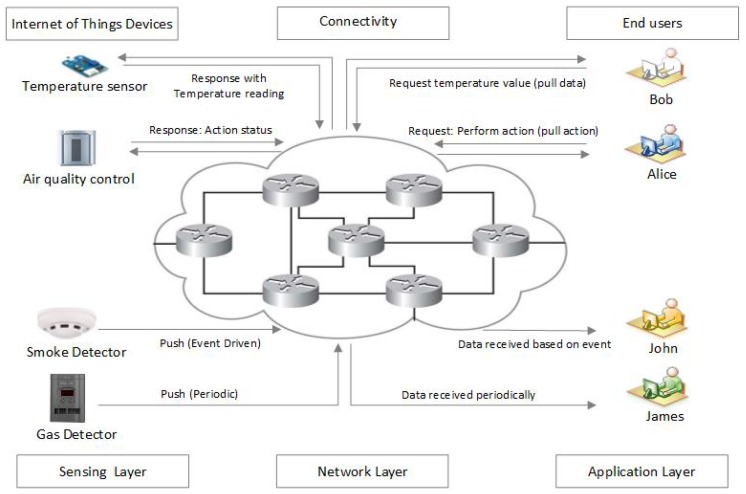
Smart building communication service models.

The motivation of this paper can be illustrated with an example of communication in a smart building. A smart building is composed of heterogenous devices with different communication protocols and applications including thermometers, the lighting system, smoke detectors, an air quality control system, an automatic door locking system, gas detectors, a heating, ventilation, and air conditioning (HVAC) system, and elevators. These heterogenous devices often produce data on a massive scale, control different peripherals in the smart building, and provide real-time feedback to end users. Communication between these devices, as well as with end users, follows different service models and can be broadly classified in 4 categories: pull (data collection), pull (action-based control commands), periodic push, and event-driven push. These service models are explained through different use cases presented in Figure 3.

### 4.1. Pull (Data Collection)

The pull (data collection) service model can be described by the following example: A user (Bob) in the smart building is interested in getting the temperature of room three on the second floor. Thus, Bob will generate a request which carries the network address information of the relevant temperature sensor. The network forwards this request to the temperature sensor, which respond with the temperature value. In this service model, the end user will always initiate the request, and the temperature sensor or destination node will only forward data in response to a request.

### 4.2. Pull (Action-Based Control Commands)

A smart building may comprise different control systems that operate on specific commands. For example, an air quality control system is installed on the second floor of a smart building, and a user (Alice) wants to turn it on. In this case, Alice will generate a request which carries the network address information of the air quality control system and the action command (for example, “turn on”). The network will forward this request by employing the network address information and, once the request reaches the destination, the air quality control system will be turned on. After executing the command, the air quality control system may respond with a Data packet that carries an acknowledgment message such as “The system has been turned on”. In this service model, an end user initiates the request, but the request carries an action command that needs to be executed at the destination node.

### 4.3. Push (Event-Driven)

In the occurrence of a particular event, especially in the case of an emergency, a sensor node or IoT device may forward an unsolicited Data packet transmission to end users or to other devices in the smart building. For instance, a smoke detector in room one on the second floor has sensed smoke in the room. Thus, it will immediately forward a Data packet to the fire alarms, as well as to the end user (John), so that precautionary action can be taken to avoid a more dangerous situation. In this service model, the end user does not generate the request but, rather, the end device or sensor node generates a Data packet in response to an event. The Data packet in this case must carry the destination network address information of the fire alarms and of John.

### 4.4. Push (Periodic)

Devices in smart buildings, or sometimes end users themselves, may require measurements from a specific device at fixed intervals, typically in minutes or hours. For example, a smart energy system in a building monitors the energy consumption of different devices in room one and periodically forwards the to a user (James). In this service model, an end device initiates communication by forwarding a Data packet which carries the destination network address information of James.

All the above-mentioned service models employ traditional IP-based solutions which are inefficient and may cause significant challenges if more and more devices are installed in the building [12]. For example, fetching data from a single sensor can require complete address information on different layers including VLAN ID, subnet, IP address, port number, and device ID [13]. As a result, managing a variety of devices and applications is a significantly complex and time-consuming task, particularly on an enterprise scale where thousands of data acquisition points may be deployed across different applications. Moreover, to overcome certain address challenges, middleware is often used to provide mapping functionality from descriptors to lower-level network addresses [13], and this creates additional configuration burden and can decrease performance.

To address these limitations, NDN can be used as an alternative because it forwards application layer names directly to the network layer and therefore mitigates the need for middleware. Moreover, the name-based forwarding mechanism in NDN eliminates the need for individual and complex network address mechanisms for each device. In this paper, we propose a name-based query framework that uses NDN as the network layer protocol to resolve the challenges of efficient heterogenous data extraction from different IoT devices in a smart building, of providing a reliable mechanism for the exchange of action-based control commands among different nodes, and of securing the communication flow between devices.

## 5. The Proposed Name-INtegrated Query (NINQ) Framework

A sensor network is a collection of resource-constrained nodes equipped with limited battery capacity, low computational power, restricted memory storage, and low-power transceivers. These nodes are deployed in an environment, such as a smart building, for monitoring, sensing, or controlling various parameters such as temperature, pressure, humidity, and air quality [44]. These nodes often generate heterogeneous and transient raw data in abundance, and the transmission of such heterogeneous content requires single or multiple hops from sensor nodes to resourceful sink nodes or to a CC. The CC or sink nodes may store the content in their memory, and, later, it can be requested by an end user. In NDN, the CRs cache the content as it is returned to the end user, thus creating a sort of distributed database in the network. The NDN architecture employs a hierarchical naming scheme to extract the content from provider nodes, which can be any sink or intermediate node that has content. This is an inefficient approach (name-only) when the content is heterogeneous, and values are scalar and updating in real time. The proposed NINQ framework provides an efficient, flexible, and expressive query mechanism to filter the content based on the consumer’s own custom logic. Moreover, the NINQ framework supports command execution on the node based on constraints.

### 5.1. Interest Packet Components in the NINQ Framework

The proposed framework divides the Interest packet into three components: (1) hierarchical name; (2) cryptographic hash of the query; and (3) the query (filtration logic and/or action command). Further detail about each component is described below.

#### 5.1.1. Name Components

**Figure 4:**
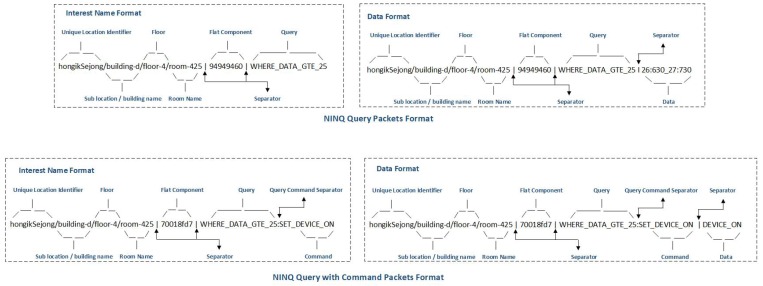
NINQ packet format.

In a NINQ, the name component is generated by combining several sub-components: (1) unique location identifier; (2) building name or sub-location identifier; (3) floor number; and (4) room number. These sub-components are joined to form the kind of hierarchical name component that is best suited for building management in, for example, a smart home, campus, or hospital. The name component is used as a prefix to locate the information retrieval site and aggregate the subsequent same requests on the router to optimize the routing table entries. Figure 4 illustrates the format of the Interest and Data packets in the proposed NINQ framework, and Table 2 summarizes the definitions of these components. A detailed description of the flat and query components now follows.

#### 5.1.2. Flat Component

To secure the query/command component, the FNV1a non-cryptographic hashing algorithm [45] is employed. The high distribution of the FNV hash function makes it well suited to hashing strings such as hostnames, filenames, URLs, and IP addresses [46]. The 32-bit FNV1a algorithm has already been employed in an NDN naming scheme [8] to generate a flat component although the purpose there was to secure the name and data components in that scheme. In the NINQ framework, it is used to maintain the integrity of the query/command component. The algorithm to compute the hash is presented in Algorithm 1. In this algorithm, all variables, such as offset_basis, FNV_Prime and octet_of_data, are unsigned integers, and their lengths are the same except for octet_of_data which is an 8-bit unsigned integer. Since the flat component is a 32-bit hash, the values of offset_basis and FNV_Prime are therefore 16,777,619 (0x01000193) and 2,166,136,261 (0x811C9DC5), respectively. The hash for a query with the command component “WHERE_TEMP.VALUE_GTE_25:SET_AC_ON” is 0x70018fd7 [47].
**Algorithm 1** FNV1a hashing algorithm*hash* ← *offset_basis*for each octet_of_data to be hashed*hash* ← *hash xor octet_of_data**hash* ← hash * FNV_Primesreturn hash

Once the hash is obtained, it is inserted between the name and query components separated by “|” in the Interest and Data packets. Whenever the CC receives an Interest, it first extracts the query component and computes its hash using the FNV1a algorithm and then compares its value with the flat component. If the hash values are equal, it will process the request and execute the query. However, if the values are not equal, the query component has been modified by an intruder, and, therefore, the CC will not execute the query. Instead, it may respond with a Data packet that contains information that a security flaw has been detected.

#### 5.1.3. Query Component

The query component begins immediately after the separator “|” with a “WHERE” keyword which denotes that the content should be filtered according to whatever logic follows it. Each subsequent element is separated by “_” and plays a key role in filtering the content. The first element is a combination of two dynamic keywords separated by “.” in which the first represents the name of a content collection (e.g., temperature or pressure) to be filtered while the second defines the field name such as ID, VALUE, or TIME. These two words are combined to form a unique key that is used to filter the collection content. For example, the element “TEMP.TIME” describes that the temperature collection should be filtered based on the time values were obtained, and “PRESR.VALUE” asks that the pressure collection be filtered according to the values in it.

The second element in the query structure is a comparison operator which plays the most important role in the filtering of the content. For example, the comparison operator “GTE”, meaning greater than or equal to, can be used to compare the value in the field name. A detailed description of all operators is presented in Table 3. The last part of the query component is a constant value which is used by the comparison operator to filter the content. When combined, the examples above could form an expressible query such as “WHERE_TEMP.TIME_GTE_10” which describes a filter for all temperature values obtained after 09:59.

A query may also return aggregated results. For example, “WHERE_TEMP.TIME _BET_9_AND _14_SELECT_AVG” would filter all content in which the temperature is obtained between 09:00 and 14:00, and the CC would then compute the average of the returned temperature values. The average value is then forwarded to the consumer as the result. Table 4 describes the interpretation of several queries although query scope is not limited to those presented; many other combinations based on IoT application requirements can also be used to define the filtration logic further.

#### 5.1.4. Command Component

A command component starts with a “SET” keyword and contains the action that needs to be performed by the device. A command may be concatenated with a constraint by using “:”. For example, “WHERE_TEMP.VALUE_BET_25_AND_50:SET_AC_ON” has both constraint and command elements separated by “:”. The syntax of the constraint is similar to a query but differs in how it works. For instance, the constraint in “WHERE_TEMP.VALUE_BET_25_AND_50:SET_AC_ON” indicates that the temperature value must be between 25 and 50 °C. The overall interpretation of this command, therefore, is to turn on the AC if the temperature in the room is between 25 and 50 °C. Most of the queries outlined in Table 4 can be used as constraints when combined with a command component. For example, “WHERE_TEMP.VALUE_LTE_20:SET_AC_OFF” represents the scenario that if the temperature is less than or equal to 20 °C, the AC will be turned off. In another example, the command “WHERE_TEMP.VALUE_GTE_25:SET_AC_OFF” is an instruction to turn off the AC if the room temperature is greater than or equal to 25 °C.

A command can also be sent without a constraint, for example, “SET_AC_OFF” or “SET_AC_16”. These commands simply turn off the AC or set the temperature to 16 °C. Table 5 presents some of the commands that can be used in a smart building, and Table 3 shows the keywords that have special meanings in the proposed NINQ framework.

### 5.2. Communication Scenarios

To analyze communication scenarios in the proposed NINQ framework, this paper uses a smart campus as a reference model. A smart campus may consist of numerous sub-buildings, each with multiple floors and rooms. Moreover, each room may be equipped with various resource-constrained sensors and control systems, such as temperature sensors and air quality or lighting control systems, and one resourceful CC (sink node) deployed at one-hop distance. These sensor or control devices perform set tasks in the room and forward the detected values, control information, and status to the CC. The CC caches the received heterogenous content in its memory so that a consumer may fetch (pull) it by sending a query-based Interest packet. The following subsection describes the workings of a simple pull mechanism when employing the NINQ framework.

#### 5.2.1. Simple Pull

**Figure 5:**
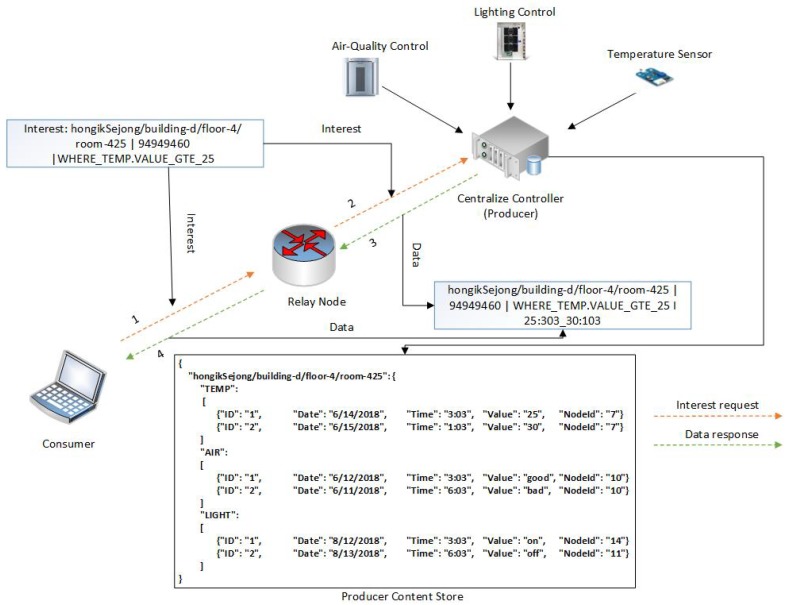
Pull service model.

Figure 5 depicts a simple pull service model and describes the workings of the proposed NINQ framework in this scenario. A consumer node issues a query-based Interest message (step 1) with the following name: “hongikSejong/building-d/ floor-4/room-425|94949460|WHERE_TEMP. VALUE_GTE_25”. The name consists of multiple hierarchical components (excluding flat and query components) that are used to locate the information retrieval site, a flat component to secure the query component, and the query component itself. In this Interest packet, the consumer node is interested in all temperature values that are greater than or equal to 25 °C. This message is forwarded by the outgoing face of the consumer node and received by the relay node at its incoming face. After receiving the Interest packet, any intermediate node with the requested content may satisfy the incoming request. However, if the relay node does not have the content, the Interest packet is forwarded via its outgoing face and received by the CC’s incoming face (step 2). Since the interest has now reached the producer node, it can be satisfied. The CC extracts the query component and compute its FNV1a hash. Once the hash value is obtained, it is compared with the flat component of the Interest packet. If these values are different, an intruder has modified the query component, and the CC will simply respond with a Data packet that contains information that a security flaw has been detected. However, if the hash values are the same, the CC will filter the data based on the logic defined in the query. The CC may then respond with the filtered content, or it may send “No results found” in the Data packet if the executed query returned an empty string. The filtered content or “No results found” message will be forwarded back to the consumer (step 3). Once this message reaches the relay node, it will be cached inside it with the query. The rationale of caching the data with the query is that if an Interest packet with the same query arrives in the future, it can be satisfied straight away without spending time to evaluate the query again. After caching, the relay node forwards the Data packet to the consumer node (step 4) and concludes the communication. To support this pull communication scenario, Algorithm 2 describes the forwarding of the Interest packet from the consumer to the producer.
**Algorithm 2** Received Interest (Query) in the Proposed NINQ**procedure**Filtering content based on query    *ContentName* ← *Substring(Hierarchical Name Component);*    *HashValue* ← *Substring(Flat Component);*    *Query* ← *Substring(Query Component);*    *ValidateQuery* = ComputeFNV1aHashAndCompare(*HashValue,Query*)    **if** (Validation Pass) **then**        *resultCollection* ← FilterContentStore(*Query*)        **if** (resultCollection is empty ) **then**           *Create PIT;*           **if** (Record Not in PIT ) **then**               *Create PIT entry with incoming and outgoing face id;*               *Initialize timer (s);*               *Forward interest using FIB;*           **else**               *Discard Interest;*           **end if**        **else**           *SendDataPacket: resultCollection*        **end if**        *SendDataPacket: ValidationFailed status code*    **end if****end procedure**

#### 5.2.2. Pull (Action-Based Control Commands)

**Figure 6:**
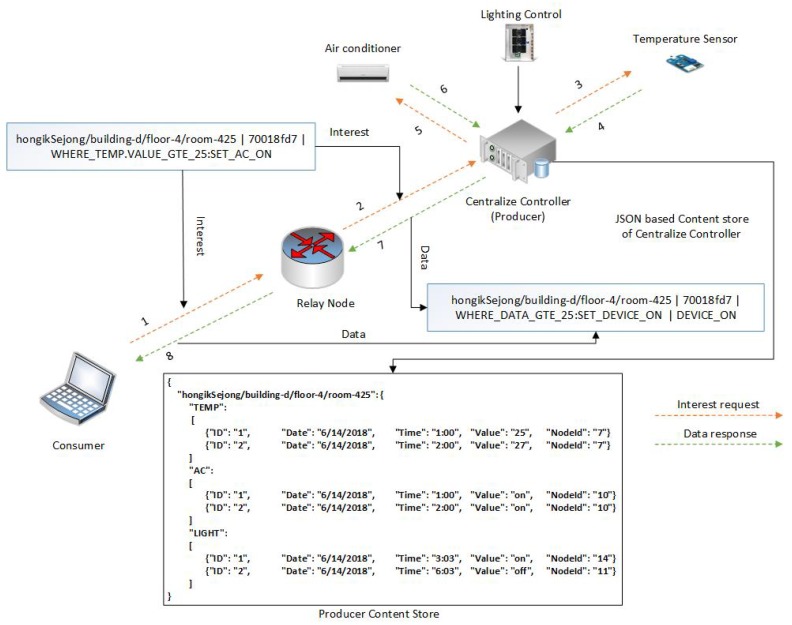
Pull (command) service model.

Figure 6 depicts a simple pull with action command service model and describes how the proposed NINQ framework works in this scenario. A consumer node issues a query-based Interest message (step 1) with the name “hongikSejong/building-d/floor-4/room-425|70018fd7| WHERE_TEMP.VALUE_GTE_25 SET_AC_ON”. In this Interest packet, the consumer node is issuing a command that the AC in room 425 must be turned on if the room temperature is greater than or equal to 25 °C. This message is forwarded from the consumer node’s outgoing face and received by the relay node at its incoming face. The intermediate node will simply forward the message (step 2) because it cannot perform the action command. Once the Interest packet reaches the producer, it can be satisfied. The CC extracts the command component and computes its FNV1a hash. The computed FNV1a will then be compared to the flat component of the Interest packet. In the case of a mismatch, the CC may respond with a Data packet containing a security flaw message. However, if the hash values are equal, the CC fetches the constraint “WHERE_TEMP.VALUE_GTE_25” and subsequently checks whether the temperature in the room is greater than or equal to 25 °C (steps 3 and 4). If the temperature is greater than or equal to 25 °C, the CC will execute the command and the AC will be turned on (steps 5 and 6). The CC can then respond with a Data packet that contains a form of acknowledgment that will be forwarded to the consumer node (steps 7 and 8). To support this communication scenario, Algorithm 3 describes the forwarding of the Interest packet from the consumer to the producer node. The command can only be satisfied by the CC.

#### 5.2.3. Push (Event-Driven or Periodic)

**Figure 7:**
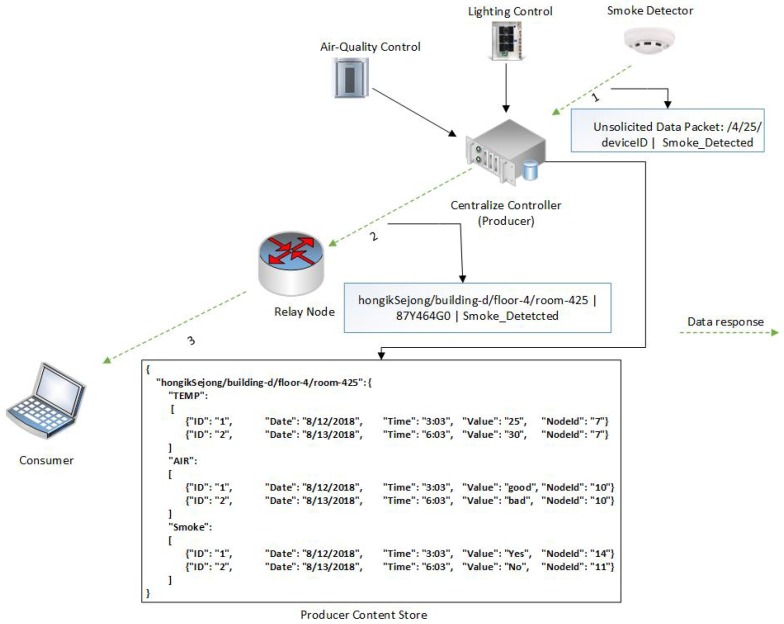
Push service model.

In a push-based service model, an end device starts the communication based either on some event or on pre-configured periodic transmissions (Figure 7). Imagine an emergency situation where smoke is detected in room 425 on the fourth floor of D building; the smoke detector immediately forwards a Data packet (hongikSejong/building-d/floor-4/room-425|87Y464G0|Smoke_Detected) to the CC installed in that room (step 1). To support the push transmission of unsolicited data, the NINQ framework modifies the existing Networking Forwarding Daemon (NFD) “forwarder” class implementation to allow unsolicited transmissions to all available node faces except that from which the Data packet was received. Since the CC is attached to a relay node, it will forward the data via the relay node’s face (step 2). As soon as the Data packet arrives at the relay node, it will repeat the same process as the CC and eventually deliver the information to the consumer (step 3). To control data broadcast in the case of wireless ad hoc or simple wired networks in which a single node is connected to multiple other nodes, the NINQ framework employs our previous work presented in [31]. In this periodic push service model, a CC may send data at intervals of, for example, four hours, although the interval may vary from application to application. An example of this type of transmission using the NINQ framework might be “hongikSejong/building-d/floor-4/room-425|70018fd7|WHERE_TEMP.TIME_BET_02-10-19-14_AND _03-11-2016-18|20,22,23,23” by which the CC will forward temperature of room 425 from 14:00 to 18:00.
**Algorithm 3** Received Interest (Command) in the Proposed NINQ**procedure**Command Execution    *ContentName* ← *Substring(Hierarchical Name Component);*    *HashValue* ← *Substring(Flat Component);*    *Constraint* ←*Substring(Constraint Component);*    *Command* ← *Substring(Command Component);*    **if** (Centralize Controller) **then**        *Validate* = ComputeFNV1aHashAndCompare*(HashValue, Constraint, Command)*        **if** (Validation Pass) **then**           *checkConstraint* ← ApplyConstraint*(Constraint)*           **if** (constraint passed) **then**               ExecuteCommand*(Command)*               **if** (command executed) **then**                   *SendDataPacket: Command Executed Successfully*               **else**                   *SendDataPacket: CommandExecutionFailed status code*               **end if**           **else**               *SendDataPacket: ContraintFailed status code*           **end if**        **else**           *SendDataPacket: ValidationFailed status code*        **end if**    **else**        *Check PIT;*        **if** (Record Not in PIT ) **then**           *Create PIT entry with incoming and outgoing face id;*           *Initialize timer (s);*           *Forward interest using FIB;*        **else**           *Discard Interest;*        **end if**    **end if****end procedure**

### 5.3. Advantages of the NINQ Framework

The NINQ framework reduces the number of transmissions in the network which results in high Interest and command satisfaction rates, reduces overall round-trip time, and eliminates the chance of congestion in the network. Moreover, and in addition to static environments, the proposed framework is well suited for networks which include mobile nodes. Before the simulation results are presented, this section will provide a logical comparison of the NINQ framework with the other recent NDN naming schemes such as HFHN or ISI.

**Figure 8:**
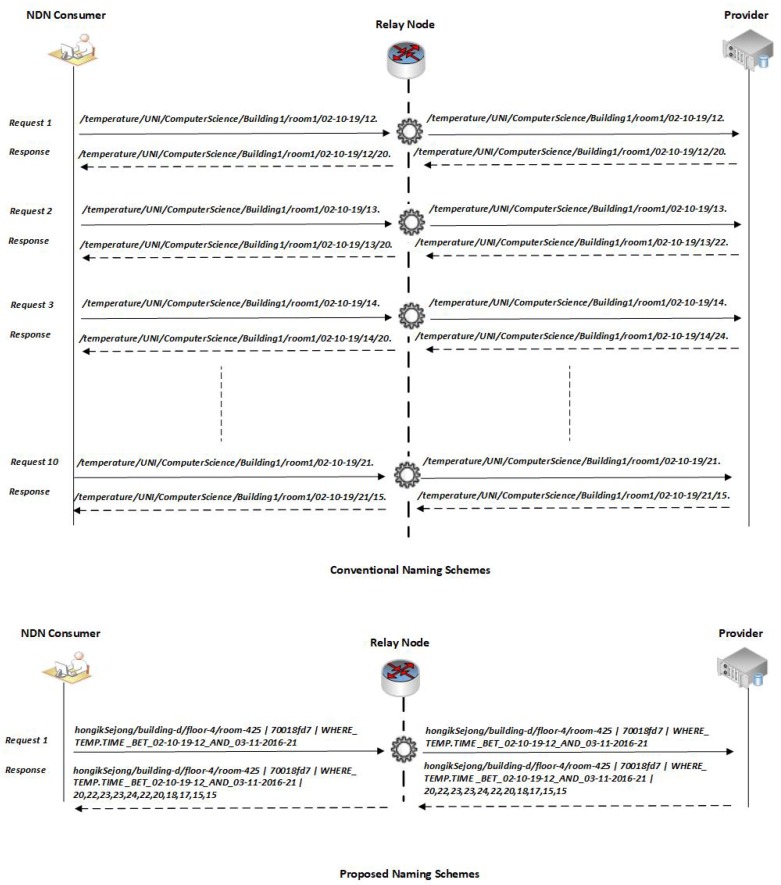
A comparison of NINQ with conventional naming scheme.

We will first use a simple pull service model example. Figure 8 depicts a scenario in which an end user is interested in getting temperature values from between 12:00 and 21:00 from the temperature sensor and CC installed in room number 425 on the fourth floor of D building at Hongik University Sejong Campus. In conventional naming scheme, the consumer will forward 10 different Interest packets to the CC. According to the solution proposed in ISI, the format of the Interest packet would be “/temperature/HongikUniversitySejong/ComputerScience/BuildingD/room425/02-10-19/12” which asks for the temperature values in room 425 at 12:00. The response to this Interest packet will be the single value recorded at 12:00 on the given date. According to this format, the consumer node must then forward nine additional packets, one each for the different time values of interest. This technique is highly inefficient because it creates numerous Interest packet transmissions in the network which may increase the chance of congestion, particularly if there are multiple consumers on the network, and any response delay could be increased because of the high request load on the CC. Moreover, if the consumer node is mobile, there is a high chance that it will be disconnected from the access point before all Interest packets have been sent. Another aspect is that the length of the requested temperature data is very short which wastes the opportunity to provide more information in the Data packet.

The NINQ scheme, on the other hand, overcomes these limitations through its efficient, expressible, and human-readable query mechanism. In the NINQ framework, if a user is interested in the same information as above, the format of the Interest packet would be “hongikSejong/building-d/floor-4/room-425|70018fd7|WHERE_TEMP.TIME_BET_02-10-19-12_AND _03-11-2016-21” which requests the temperature values from the CC for the period between 12:00 and 22:00 on the given date. Using this format, only one Interest packet transmission is required because it will fetch all values within the requested range.

Moving to a pull (action-based commands) service model example, an end user is interested in configuring the AC unit in room 425 based on the temperature in that room. For this configuration, the consumer requires temperature readings from the CC from the morning to the evening, for example, from 08:00 to 18:00. In conventional naming schemes, the consumer node must fetch each reading from the CC and then forward the AC configuration command to the CC. As such, two major tasks are being performed: (1) the consumer is fetching 11 different temperature readings from the CC by sending 11 different Interest packets; (2) the consumer must analyze the returned data, for example by computing an average, and forward the resulting AC configuration command. Across these two tasks, the consumer node sends 12 requests to fulfil the requirement.

In contrast, the entire process can be completed with a single NINQ request. The NINQ Interest and Data packets for the above example would be “hongikSejong/building-d/floor-4/room-425|70018fd7| WHERE_TEMP.TIME_BET_9_AND_14_SELECT_AVG_EQ_21 SET_AC_18” and “hongikSejong/ building-d/floor4/room425|70018fd7|WHERE_TEMP.TIME_BET_9_AND_14_SELECT_AVG_EQ_21 SET_AC_18 Processed”, respectively.

## 6. Performance Evaluation

This section will discuss the performance evaluation of the proposed NINQ framework by considering communication scenarios at a smart campus.

### 6.1. Simulation Environment

**Figure 9:**
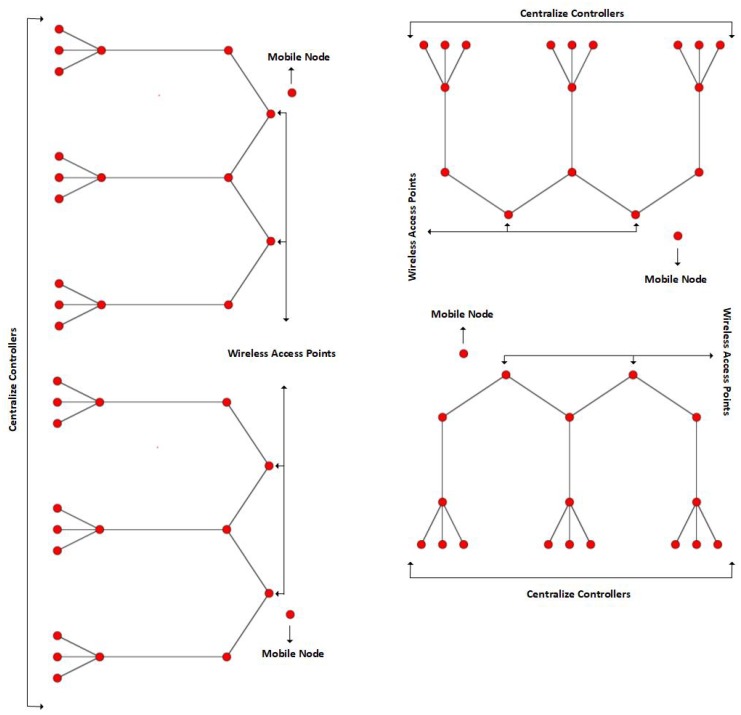
Simulation environment.

We consider multiple buildings on a smart campus, each with numerous floors and rooms. Each room is equipped with a CC which is connected to temperature and smoke sensors and an AC system. Each building has two wireless access points (WAPs) connected to the various CCs which also provide wireless connection to the mobile nodes within each building. The simulation scenario is presented in Figure 9, and the NINQ framework was simulated using ndnSIM [48] on Linux Ubuntu. A Core i7 PC with 8 GB RAM was used for both implementation and performance evaluation. The NFD and ndnSIM codebase were modified in accordance with the requirements of the proposed framework. For the WAPs, a constant position mobility model was employed with different *x*- and *y*-axis. For mobile consumer nodes, a constant velocity mobility model was used. The mobile nodes in buildings on the left are moving vertically while those on the right-hand side are moving horizontally (Figure 9). We vary the speed of the mobile nodes to analyze the behavior of the proposed framework, although the number of nodes employed in the simulation is fixed. However, the NINQ framework is scalable and can be validated for use on a large scale. The remaining simulation parameters are summarized in Table 6.

### 6.2. Results and Discussion

The following performance metrics were used to evaluate the effectiveness of the NINQ framework. We compare our framework with the recent HFHN and ISI schemes.

#### 6.2.1. Interest Satisfaction Rate

**Figure 10:**
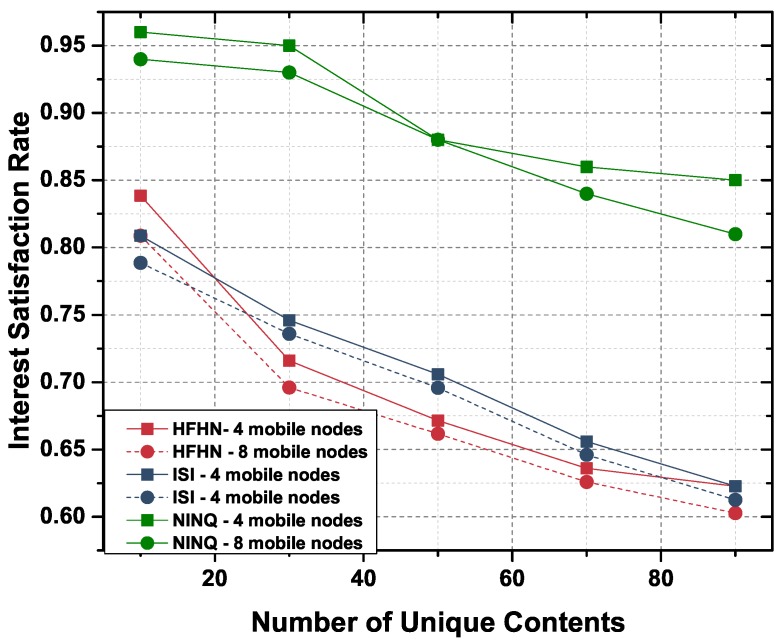
ISR in a network by number of unique contents.

The ISR in a network is the ratio of total Interests satisfied to total Interests generated. Figure 10 shows a performance comparison in terms of satisfied Interests that the NINQ approach achieved as compared to the HFHN and ISI schemes. Two rounds of simulation were performed for all three schemes, with four mobile consumers in the first and eight in the second. The speed of all mobile nodes was constant throughout each simulation. For more in-depth analysis of the ISR, we then varied the number of requested unique contents from 10 to 90 at intervals of 20. From these results, we find that the NINQ approach achieves significantly better ISR in comparison to the HFHN and ISI schemes. The reason for this is that a single Interest packet using the NINQ framework can fetch multiple unique contents in a single Data packet by defining the custom logic in its query component. In contrast, neither HFHN or ISI include a way of retrieving multiple contents collectively (as discussed earlier in Section 5.3) and each forwards multiple single Interest packets which can cause congestion in the network, hence reducing the ISR.

**Figure 11:**
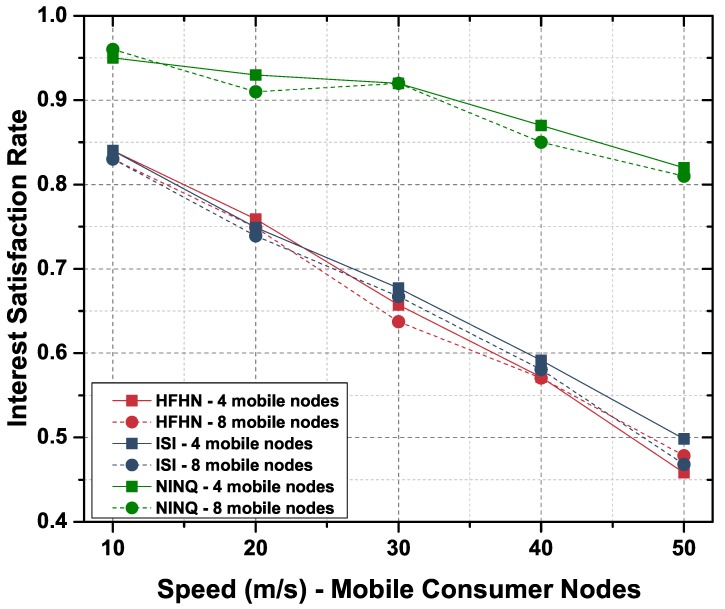
ISR in a network by speed of mobile nodes.

Another reason for the low ISR values is the presence of mobile consumer nodes in the network. To analyze the impact of mobility on the ISR, we performed separate simulation by varying the speed of the mobile nodes. A simple constant velocity mobility model was employed, and we adjusted the speed from 10 to 50 m/s. A constant number of unique contents were requested during the connection period. It is evident from the results presented in Figure 11 that the NINQ scheme outperforms HFHN and ISI. This is possibly because when nodes are mobile, they may become disconnected from the WAP before sending all requests for the 25 unique contents. Conversely, it is highly likely that the single request required in a NINQ will fetch the number of unique contents within the connection period.

#### 6.2.2. Command Satisfaction Rate

The command satisfaction rate (ISR) of a network is the ratio of total commands satisfied to total commands generated. In the HFHN and ISI frameworks, particular Interest packets only contain a command component because these schemes first fetch data from the producers, analyze it through constraint processing, and then forward the resulting command to the producer to perform the appropriate action. In this scenario, multiple requests are transmitted across the network before the actual command is sent. In contrast, the NINQ framework Interest packets simultaneously carry constraint and command components, the constraint component eliminating the need for multiple packets.

**Figure 12:**
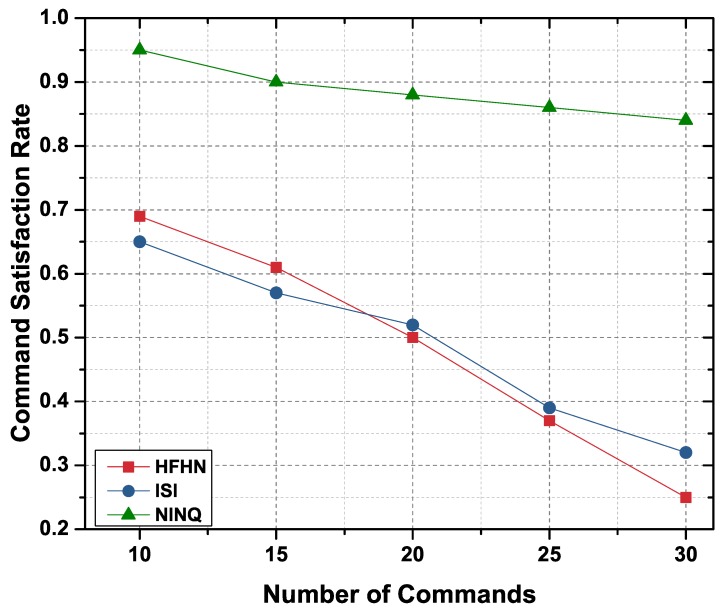
CSR in a network by number of commands.

Figure 12 compares scheme performance in terms of satisfied commands for the NINQ, HFHN, and ISI naming schemes. Similar to the ISR results, the NINQ approach achieved a higher CSR than the other schemes. The reason for this is that to forward, for example, 15 commands in HFHN and ISI, it is first necessary to send hundreds of Interest packets. However, since a NINQ eliminates the need for these huge numbers of packets, congestion can be avoided, and the nodes will receive a response within the connection period.

#### 6.2.3. Number of Packets Processed

In our simulation for number of packets processed in the network, it was assumed that the producer node contains temperature sensor readings recorded every 15 min from which it creates a CS of multiple Data packet collections. In HFHN or ISI, if a consumer wishes to fetch these packets from the producer according to the time they were recorded, multiple separate Interest packets must be sent to the producer, and so if a CS contains many different Data packets, each with a different time value, the consumer node needs to forward a correspondingly large number of Interest packets. On the other hand, in a NINQ, a small number of Interest packets can fetch multiple recordings by defining an appropriate query.

**Figure 13:**
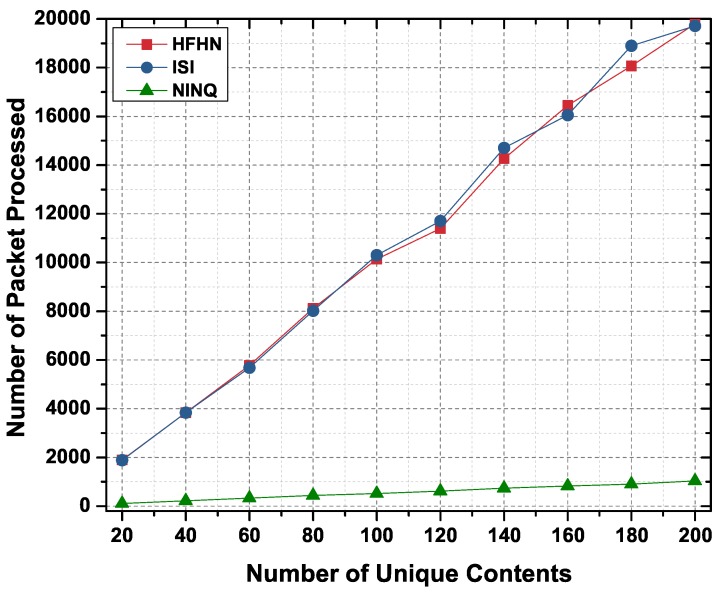
Number of packets processed in a network by number of unique content objects.

Figure 13 shows the processing simulation results in which the average number of transmissions in the HFHN and ISI schemes are higher compared to the proposed method because a separate request is issued for each Data packet and the intermediate nodes regenerate each one. However, in the NINQ approach, the number of transmissions is relatively small because a single Interest packet is enough to fetch multiple sensor readings in a single Data packet. Since the number of transmissions is very small, the NINQ framework avoids the risk of network congestion and reduces the latency that can occur due to large numbers of packet transmissions. Moreover, the NINQ framework also uses a small amount of bandwidth in comparison to the other two naming schemes.

#### 6.2.4. Energy Consumption

**Figure 14:**
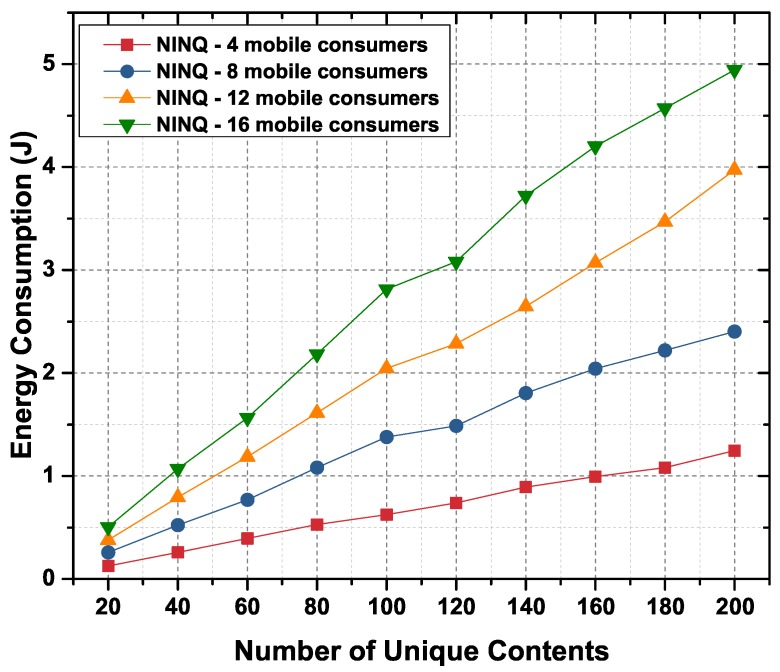
Energy consumption in an NINQ-based network as function of the number of unique content objects.

It is evident from previous results and discussion that the NINQ framework outperforms both the HFHN and ISI schemes in terms of packet processing. The proposed framework minimizes the number of Interest and Data packet transmissions in the network and maintains a smaller number of PIT entries on the forwarding node. As a result, NINQ minimize transmission energy and save battery resources. Instead of comparing the energy consumption of the NINQ framework with the HFHN and ISI schemes, we therefore only consider the proposed framework with varying numbers of mobile consumer nodes. Figure 14 shows the total amount of energy consumed by all nodes (static and mobile) for different numbers of unique content objects. Energy consumption is directly proportional to the number of requests for unique content objects forwarded in the network. However, the nodes that participate in the execution of a query consume more energy than those that only forward the Interest or Data packets. Thus, the total amount of energy consumed in the NINQ-based network is the sum of the energy consumed in forwarding an Interest packet, in executing the query, and in forwarding the Data packet back to the mobile consumers. As shown in the Figure 14, as the number of mobile nodes increases in the network, energy consumption also increases. The reason for this is that each mobile consumer forwards an equal number of requests across the network.

**Figure 15:**
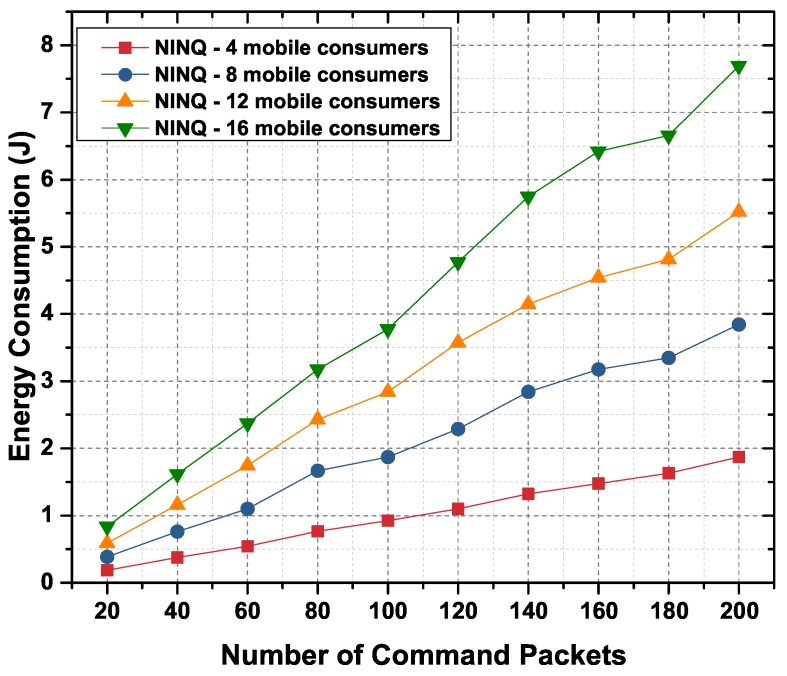
Energy consumption in an NINQ-based network as function of the number of commands.

Figure 15 shows the total amount of energy consumed by all nodes (static and mobile) when the consumer nodes are forwarding commands in the network. Comparing the results shown Figure 14 and Figure 15, it is evident that the network consumes more energy when it is processing commands. The total amount of energy by commands is the sum of energy consumed in forwarding an Interest and Data packet, in applying the constraint, and in executing the command.

#### 6.2.5. Average Delay

**Figure 16:**
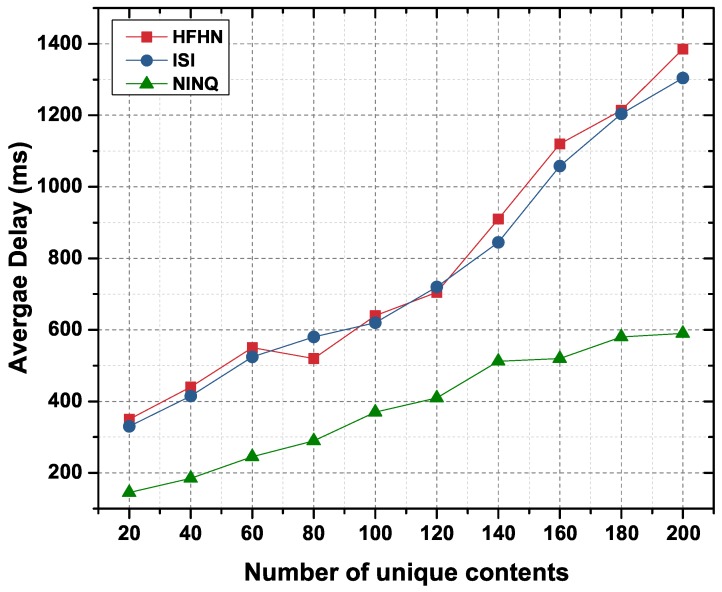
Average delay in a network as function of number of unique contents.

**Figure 17:**
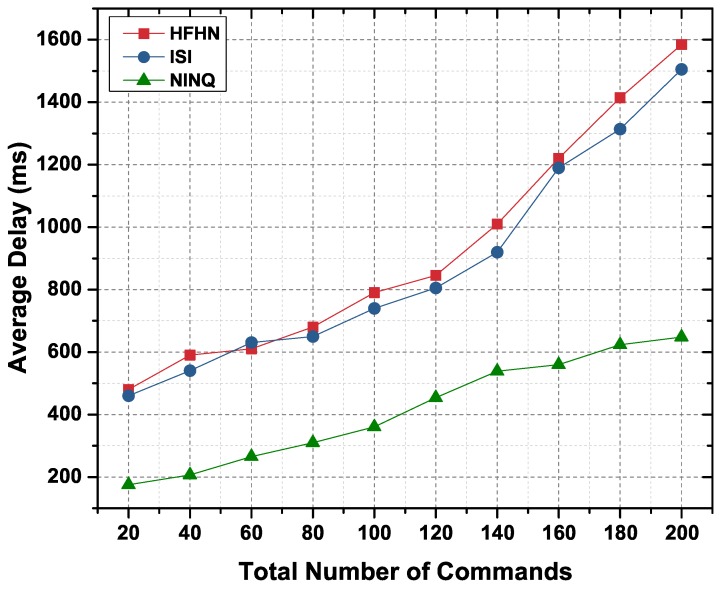
Average delay in a network as function of total number of commands.

Average delay is the sum of the time it takes for an Interest packet to reach the CC or provider node, to process the query/command on a node, and for the Data packet from the CC or provider to reach the consumer node. Figure 16 presents the average delay for varying numbers of requests for unique contents. Since, in the case of the HFHN and ISI schemes, multiple requests are forwarded to fetch all unique contents, the average delay is significantly higher as compared to that in the proposed NINQ framework. The average delay for NINQ-based command execution (Figure 17) is slightly higher than the average delay for NINQ queries (Figure 16). The reason here is that there is an additional delay in command execution on a CC.

## 7. Future Directions

The NINQ named-based query framework proposed here provides flexibility for the end user to filter raw data from IoT devices based on their own custom logic. In addition, an end user or device can express an action command with or without constraints. The proposed framework is expressible, human-readable, and implements a secure query mechanism. However, this is only the first step towards query-based NDN for IoT infrastructure with following several important areas yet to be explored.

### 7.1. NINQ Testbed Implementation

To derive practical benefits from the NINQ framework, there is a need to fully analyze and deploy the proposed scheme on a real testbed. To do, the NINQ codebase could be deployed on the RIOT operating system [49]. The authors of [50] provide design and implementation details of the NDN protocol stack for RIOT. However, deployment of this stack with the NINQ codebase on supported hardware such as Arduino Mega2560, STM32 Nucleo32-F303, or RE-Mote will be a challenging task that will require much time and research effort.

### 7.2. Multiple Query Execution

Since the CC is attached to multiple sensing and controlling units, it may be possible that the end user is interested in filtering content of different kinds, for example, temperature, pressure, and air quality data. To filter such content, an end user may send an Interest packet with multiple queries. However, to support the transmission of longer packets and the temporally complex execution of multiple queries requires further research.

### 7.3. Query Execution Plan: Evaluation and Time Complexity

To optimize query execution, modern databases such as SQL and MongoDB employ execution plans that use a query optimizer. In the proposed framework, a query execution plan could be a set of sequences or steps that the CC performs to execute the query. A need to devise a proper query plan for the NINQ scheme arises because the end user formulates their query using filtering logic but does not tell the CC the exact order in which to execute it. Currently, there is no managed way for a query execution plan, and so a proper query planner code module must be developed. The responsibility of this type of module is to fetch the best query execution plan to efficiently filter the raw content. Relatedly, there is a need to analyze the time complexity of filtering raw data and executing action-based commands.

### 7.4. Augmented Reality and Edge Support

In the 5G communication environment, and beyond, demand for augmented reality (AR) services is expected to increase. In AR, real-time feedback for the end user is required and, to fulfil this requirement, there is a need to process huge amounts of data very quickly. To support these kinds of services, edge computing is a promising concept in which highly powerful devices are placed near the end user to process large data quickly and provide this real-time feedback [51,52]. There is a need to further explore the proposed NINQ framework within the edge computing paradigm to potentially support AR-based services.

### 7.5. NINQ-Lite for Wireless Sensor Networks

A WSN comprises a set of nodes equipped with limited resources such as low computational power, constrained battery capacity, small memory size, and low-power transceivers. Consequently, the maximum transmission unit (MTU) in WSNs is relatively very small at 127 bytes, as compared to an MTU of 1280 bytes in devices on the Internet [34]. This limits the applicability of the proposed framework in WSNs because it could not forward an Interest packet containing multiple filtration logics. However, through further research, a lighter version of NINQ (NINQ-lite) may be developed for WSNs which would aim to fit the query logic within a 127-byte MTU.

### 7.6. Software-Defined Networking Interoperability

As the MTU of resource-constrained devices such as temperature and pressure sensors is small, the protocols that run on them may be different to those that run on Internet devices. Thus, it could be difficult, or impossible, to have direct communication between sensors and Internet devices. However, indirect communication using protocol translation could be used. Software-defined networking (SDN) is an emerging future Internet paradigm which supports interoperability between devices that run different protocols. Further research in this area could solve the problem of NINQ and NINQ-Lite protocol translation.

### 7.7. Multiple Chunks Analysis (Wireless)

In a NINQ, there is a possibility that a single query will return a large amount of data that may not fit in a single Data packet, particularly in wireless communication scenarios. In this case, there would be a need to divide the content into chunks and send these back to the end user one by one. On their way back to the end user, these chunks would be cached in the intermediate nodes. Since the end result is based on the custom filtering logic, a proper caching strategy is needed to store the most relevant information either with the full query logic or a sub-component of it.

### 7.8. Efficient Caching

Since Data packets in the proposed NINQ framework contain content filtered by logic defined in the query component, it is necessary to consider the following points at the time of caching: (1) The query must be cached in the intermediate CS along with the content so that if the same query arrives in the future for the same CC, the result can be provided from the intermediate node rather than from the CC; (2) The content of smart building networks and, more generally, on the IoT is transient in nature and the cached content should therefore be purged from the CS over time. This second point is highly dependent on specific application requirements.

### 7.9. DDOS Attack Due to Unsolicited Data

To support a push-based communication model, the NINQ framework allows unsolicited data to be processed across the network. However, permitting unsolicited data can cause DDOS attacks in which a malicious node frequently sends raw data to waste the energy of legitimate nodes. As such, there is a need to devise an effective solution that supports unsolicited data transmission as well as avoids DDOS attacks against the network.

## 8. Conclusions

In this paper, we argue that the queries should be forwarded on the network layer in addition to the names. Thus, we proposed a named-based query and command mechanism named NINQ framework. The position of this paper is manifold. First, an overview of NDN and IoT is presented. Second, we presented the most relevant and recent naming schemes proposed under the umbrella of NDN. Third, we described 4 different communication service models in the smart building. Fourth, a detailed explanation of the components of proposed naming scheme along with different communication scenarios are presented. Here, we argued that conventional hierarchical or hybrid NDN naming schemes are inefficient to extract the content that is transient in nature. To support our claim, we presented a logical comparison of proposed NINQ and other naming schemes. Fifth, in addition to the logical comparison we also showed our simulation results and discussed all the results in detail. It is evident from the results that proposed NINQ framework outperform the most recent naming schemes in terms of ISR, CSR, number of packets processed in the network, energy consumption, and average delay. Sixth, open research challenges and future directions of our proposed NINQ framework are presented.

## Figures and Tables

**Table 1:** Description of Packet Components.

Naming Scheme	HC	FC	QC	Pull Support	Push Support	Mobility
Naming scheme for smart building: HFHN [8]	✔	✔	✘	✔	✔	✔
Naming scheme for smart home: Marica et al. [30]	✔	✘	✘	✔	✔	✘
Naming scheme for smart building: Rehmat et al. [31]	✔	✘	✘	✘	✔	✘
Naming scheme for VANET: Bouk et al. [37]	✔	✔	✘	✔	✘	✔
Naming Scheme for UWSN: Bouk et al. [32]	✔	✘	✘	✔	✘	✘
Naming scheme for smart building: ISI [34]	✔	✘	✘	✔	✔	✔
Naming scheme for Edge computation [33]	✔	✘	✘	✘	✘	✔
Proposed Naming Scheme for smart building	✔	✔	✔	✔	✔	✔

**Table 2:** Description of Packet Components.

Component	Description
Unique Location identifier	This sub-component is used as a unique domain to identify the location
Sub-Location identifier	This sub-component is used to define the regions inside a building
Floor Number	This component is used to locate the floor in a building
Room Number	This component is used to locate the room number on some floor
Flat Component (HMAC)	FNV1a hash of query or query with command component
Query	This component is used to filter the content inside a namespace
Command	This component is used to perform action which may base on the query
Data	This component represents the filtered data obtained by executing the query
Separator (|)	Separator is used to separate the hierarchical, flat, query/command, and data.
Separator (:)	This separator is used to separate query from command

**Table 3:** Description of Keywords.

Keywords	Description
GT	Greater Than: Values in a collection must be greater the value written
	on right side of “GT” operator
GTE	Greater Than Equal: Values in a collection must be greater than or equal
	to the value written on right side of “GTE” operator
LT	Less Than: Values in a collection must be less the value written
	on right side of “LT” operator
LTE	Less Than Equal: Values in a collection must be less than or equal to the
	value written on right side of “LTE” operator
EQ	Equal: Values in a collection must be equal to the value written on right
	side of “EQ” operator
NEQ	Not Equal: Values in a collection must not be equal to the value written on
	right side of “NEQ” operator
BET	Between: Values in a collection must be in between to the values written on
	right side of “BET” operator
IN	Include: Check whether collection contain the value written on right
	side of “IN” operator
LIMIT	Limit the results to the number written on the right side of “LIMIT” operator
ASC, DESC	Ascending and Descending order
AVG, MIN, MAX, COUNT, SUM	Aggregate functions

**Table 4:** Description of Keywords.

Queries	Description
WHERE_TEMP.VALUE_GT_25	Select temperature sub-collection in which values are
	greater than to 25
WHERE_TEMP.VALUE_GTE_25	Select temperature sub-collection in which values are
	greater than or equal to 25
WHERE_TEMP.VALUE_LT_25	Select temperature sub-collection in which values are
	less than 25
WHERE_TEMP.VALUE_LTE_25	Select temperature sub-collection in which values are
	less than or equal to 25
WHERE_TEMP.VALUE_EQ_25	Select temperature sub-collection in which values are
	equal to 25
WHERE_TEMP.VALUE_NEQ_25	Select temperature sub-collection in which values are
	not equal to 25
WHERE_TEMP.VALUE_IN_25	Check whether the temperature collection have value
	25 or not. Result will contain a Boolean value.
WHERE_TEMP.VALUE_BET_25_AND_50	Select temperature sub-collection in which values are
	between 25 and 50
WHERE_TEMP.VALUE_GT_25_LIMIT_10_DESC	Select top 10 (descending order) temperature sub
	collection in which values are greater than 25
WHERE_TEMP.VALUE_GT_25_LIMIT_10_ASC	Select top 10 (ascending order) temperature sub
	collection in which values are greater than 25
WHERE_TEMP.VALUE_GT_25_SELECT_COUNT	Get the number of temperature sub collection in which
	values are greater than 25
WHERE_TEMP.TIME_BET_9_AND_14_SELECT_AVG	Get average temperature values of current day for time
	between 9 a.m. and 2 p.m.
WHERE_TEMP.TIME_BET_9_AND_14_SELECT_MIN	Get minimum temperature value of current day for time
	between 9 a.m. and 2 p.m.
WHERE_TEMP.TIME_BET_9_AND_14_SELECT_MAX	Get maximum temperature value of current day for time
	between 9 a.m. and 2 p.m.
WHERE_TEMP.TIME_BET_9_AND_14_SELECT_SUM	Get sum of temperature values of current day for time
	between 9 a.m. and 2 p.m.

**Table 5:** Example of NINQ Commands with Constraints.

Queries	Description
SET_AC_ON	Turn on the AC
SET_AC_16	Set the temperature value of AC to 16
WHERE_TEMP.VALUE_LTE_20:SET_AC_OFF	Turn of the AC if temperature is less than or equal to 20
WHERE_TEMP.VALUE_BET_25_AND_50SET_AC_ON	Turn on AC if temperature is between 25 and 50
WHERE_TEMP.VALUE_LTE_20:SET_AC_OFF	Turn off AC if temperature is less than or equal to 25

**Table 6:** Simulation Parameters.

Parameter	Value
Simulator	NS-3 (ndnSIM 2.5)
Communication Stack	NDN
NDN	Constant Speed Propagation Delay Model
Propagation Loss Model	Range Propagation Loss Model
Mobility Model	Constant Position Mobility Model , Constant Velocity Mobility Model
Simulator	NS-3 (ndnSIM 2.5)
Number of Static Nodes	68
Number of Mobile Consumer Nodes	4, 8, 12, 16
Caching Policy	LCE
Replacement Policy	LRU
Content Store Size	1000
PIT Timer	4 s
Simulation Time	1800 s
